# A road map for the management of a pregnancy complicated by maternal bladder exstrophy

**DOI:** 10.1186/s12884-024-06316-2

**Published:** 2024-03-12

**Authors:** A Hosiani, M.E. Smet, R Nayyar

**Affiliations:** 1 Department of Obstetrics and Gynaecology Blacktown Hospital, Blacktown, Australia; 2 Westmead Institute for Maternal Fetal Medicine, Westmead, Australia

**Keywords:** Bladder exstrophy, Preterm birth, Caesarean section

## Abstract

Bladder exstrophy (BE) is a congenital genito-urinary malformation where there is a defect in the abdominal wall resulting in a protruding open bladder with exposed mucosa (Resnik R.P. et al. Creasy and Resnik’s maternal-fetal medicine: principles and practice. Elsevier, 2019). Several reconstructive procedures are required to correct the anomalies, resulting in an ileal conduit which is an alternate urinary reservoir reconstructed from the terminal ileum (Madersbacher S, et al. J Urol 169(3):985–90, 2003). We describe the care of a pregnant woman with BE and outline the principles of management of her pregnancy with a multidisciplinary team. Timely pre-operative planning is advised to minimise intraoperative complications in the event of a caesarean section. The woman went on to have an uncomplicated classical caesarean section at term by midline laparotomy with a good outcome for both mother and baby.

## Background

Bladder exstrophy (BE) is part of the epispadias-exstrophy complex that results in a congenital malformation of the abdominal wall and urogenital tract [1–3]. BE is a rare anomaly with a prevalence of 1 in 400,000 births and is four times more prevalent in females than males [3]. The condition causes the bladder to evert through an abdominal wall defect with an associated abnormally shaped pelvis and possible Mullerian duct anomalies [2, 3]. Advances in surgical techniques, antimicrobial therapies and access to medical care have improved reproductive health in women affected with BE [3]. The antenatal complications include miscarriage, pelvic organ prolapse [4, 5], recurrent urinary tract infections (UTI’s), pyelonephritis and preterm birth [6]. Women with BE may also have abdominal and pelvic adhesions following multiple prior reconstructive procedures which can complicate access at the time of caesarean section (CS) [4]. Although recommended by several authors [7–9], robust evidence to support a caesearean section over vaginal delivery in preventing adverse outcomes [10] is lacking and may be due to the low prevalence of the condition.

## Case presentation

A 32-year-old primigravida woman was first seen at our hospital at 10 weeks’ gestation following a natural conception. She had a history of congenital BE that had been surgically corrected overseas. The woman had a neobladder with a urethral stoma in the right lower abdomen and performed twice daily clean intermittent self-catheterisation. She had recently been diagnosed with antibody Ro/La-negative rheumatoid arthritis which was managed with hydroxychloroquine 200 mg daily and prednisone 5 mg daily.

The pregnancy was managed by a multidisciplinary team (MDT), involving maternal fetal medicine (MFM) specialists, urologists and anaesthetists. Antenatal care included blood pressure monitoring and monthly urine midstream analysis. The woman had frequent assessment of her serum electrolytes as an ileocaecal pouch had been used to create the neobladder. A single episode of asymptomatic E. coli- UTI at 36 weeks’ was treated appropriately.

A cell-free fetal DNA test reported a low probability for trisomy 13, 18 and 21. Serial fetal ultrasounds performed during pregnancy, reported normal morphology, biometry, liquor and doppler studies. The placenta was implanted posteriorly in the upper uterine segment. During the course of the pregnancy, the patient presented four times with reduced foetal movements and was assessed as per the New South Wales health guidelines [11] with a cardiotocography, feto-maternal hemorrhage quantification and growth ultrasound.

The woman was counselled regarding the mode of delivery and a caesarean section was advised. Although open to debate, this decision was based on our clinical assessment of the maternal pelvis and advised by the urologist who had performed her reconstructive procedures. Informed consent was obtained regarding the risks of caesarean section and the impact of a classical caesarean section on subsequent pregnancies. The option of vaginal delivery was discussed with the potential risk both of cephalopelvic disproportion and subsequent (refractory) uterovaginal prolapse.

As part of the surgical planning a maternal pelvic magnetic resonance imaging (MRI) at 36-weeks’ gestation was performed and aided in determining the location of the gravid uterus in relation to the neobladder. The MRI demonstrated a conduit in the right iliac fossa, anterior and to the right of the uterus.

At 36 weeks and 5 days gestation, the decision for an elective caesarean section under general anaesthesia was taken, following recurrent episodes of decreased foetal movements. A midline laparotomy was performed to avoid the neobladder. The neobladder was identified and noted to be clear of the uterus, with no adhesions. A classical caesarean section was performed, and a live infant was delivered in good condition with an uncomplicated third stage. A pouchoscopy was performed through a flexible pyeloscope inserted into the neobladder and revealed no injury or leakage. The woman had an unremarkable recovery, and she was discharged three days postpartum.

## Outcome and follow up

A health baby was born with APGAR’s of 3, 8 and 8 at 1, 5 and 10 min weighing 2.6 kg. Contraception was discussed and an interpregnancy interval of 2-years was recommended.

## Discussion

The objective of our case report is to discuss the management of a pregnancy in a woman with BE. Although BE is rare, good antenatal care and delivery planning has the potential to minimise the risk of complications [7]. A literature review was undertaken to find similar published cases using MEDLINE, Embase and Ovid and the search terms included ‘bladder exstrophy’, ‘pregnancy’ or ‘epispadias-exstrophy complex’.

The antenatal complications in women with BE include miscarriage, preterm birth, UTI, urinary retention, pyelonephritis and uterine prolapse [4–6, 12]. Urinary retention and subsequent UTI occur possibly due to kinking of the conduit by the gravid uterus [13]. Deans et al. 2012 recommended baseline urine microscopy, culture, and sensitivity (MCS) followed by serial monitoring of urine cultures as asymptomatic bacteria is common in these women. Treatment is advised if the woman is symptomatic, new pathogens are identified or an increased colony count from baseline levels. Nevertheless, our patient was treated with oral antibiotics for asymptomatic E.Coli bacteruria as her regular urine MCS were negative until 36 weeks’ gestation. Dap et al. 2011 followed 6 pregnancies in 3 BE patients and reported pyelonephritis in 2 of the pregnancies of which one woman delivered preterm [12]. They also documented three of the six pregnancies in this cohort had malpresentations likely attributed to Mullerian anomalies.

Monthly serum electrolytes were monitored given the risk of metabolic acidosis. This occurs as the anion exchange pumps on the colonic mucosa, within the conduit exchange bicarbonate from the urine with chloride, resulting in metabolic acidosis [14].

Grion et al. observed four pregnancies that resulted in miscarriage in the context of a pelvic organ prolapse [15]. Women with BE are at increased risk of pelvic organ prolapse possibly related to the congenital weakness of the cardinal and uterosacral ligaments and associated pubic diastasis [16]. Matthew et al. reported a pregnancy with uterine prolapse which required surgical repair after failure of conservative management with a pessary [6]. This prolapse was subsequently repaired by a Restorelle mesh.

Bonner & Mohammed 2018 were the only authors who reported the use of an abdominal MRI in the third trimester to determine the location of vital structures within the abdominal cavity with respect to the gravid uterus to assist in surgical planning [4]. We performed an abdominal MRI at 36 weeks’ gestation to assist in surgical planning and minimise the risk of injury to the neobladder.

The most common surgical complications that can arise are damage to the conduit or bowel perforation [6, 10, 13, 15]. Deans et al. additionally reported an intraoperative transection of a ureter [5]. Rubenwolf et al. 2016 as well as Sharma 1998 highlighted the importance of the urologist and preferably the urologist involved in the primary care to be present at the caesarean Sects. [7, 17]. In our patient this was not possible as the reconstructive surgeries were performed overseas. Nevertheless, the primary urologist was closely involved in the planning of the caesarean section through telehealth consultation.

Several authors recommended a planned CS to avoid difficult abdominal access in an emergency and to potentially protect future continence and avoid pelvic organ prolapse that may occur following vaginal delivery [5, 6, 12, 13, 15]. Deans et al. reported on an emergency CS whereby a delay in accessing the uterine cavity resulted in a stillbirth [5]. Grion et al. 2011 identified 14 women who had a total of 22 pregnancies [15]. Only one out of these 14 women delivered by vaginal route, which involved an uncomplicated preterm delivery. Dy et al. 2015 suggested that a vaginal delivery may be considered depending on the number of previous abdominal and pelvic surgeries [13]. It is likely that this subgroup of women may represent the less severe end of the spectrum of BE. The decision regarding the mode of delivery should be individualised balancing the risk of both vaginal delivery and caesarean section and considering the woman’s wishes.

## Conclusion

Maternal BE requires tertiary level care during pregnancy. A multidisciplinary approach is ideal to ensure both good obstetric and newborn outcomes along with conserving the function and anatomy of the neobladder and conduit. A timely discussion regarding the mode of delivery as well as a meticulous pre-operative planning is beneficial in mitigating potential adverse outcomes for the mother and baby. Based on the complexity of the previous corrective procedures, the option of a preoperative MRI and a planned caesarean section may be considered. These strategies may have the potential to prevent intraoperative surgical complications, an emergency operative delivery and preserve pelvic floor integrity.

Take-Home Messages:

- MDT to provide care in a tertiary hospital for women with BE.
- Abdominal MRI in the third trimester may assist in surgical planning.
- Planned elective CS has the potential to minimise both maternal and neonatal morbidity and mortality.
- Consideration regarding the abdominal incision should take into account both the site of the neobladder and conduit to prevent surgical complications.

## Data Availability

The datasets generated and/or analysed during the current study are not publicly available due to this being a case report and no specific data has been collected for this particular publication. But we are able to provide any specific material from the corresponding author on reasonable request.
